# Assessment of reliability and validity of the 5-scale grading system of the point-of-care immunoassay for tear matrix metalloproteinase-9

**DOI:** 10.1038/s41598-021-92020-6

**Published:** 2021-06-11

**Authors:** Minjeong Kim, Ja Young Oh, Seon Ha Bae, Seung Hyeun Lee, Won Jun Lee, Yeoun Sook Chun, Kyoung Woo Kim

**Affiliations:** Department of Ophthalmology, Chung-Ang University College of Medicine, 102 Heukseok-ro, Dongjak-gu, Seoul, 06973 Republic of Korea

**Keywords:** Diagnostic markers, Corneal diseases

## Abstract

We evaluated the reliability and validity of the 5-scale grading system to interpret the point-of-care immunoassay for tear matrix metalloproteinase (MMP)-9. Six observers graded red bands of photographs of the readout window in MMP-9 immunoassay kit (InflammaDry) two times with 2-week interval based on the 5-scale grading system (i.e. grade 0–4). Interobserver and intraobserver reliability were evaluated using intraclass correlation coefficients. The interobserver agreements were analyzed according to the severity of tear MMP-9 expression. To validate the system, a concentration calibration curve was made using MMP-9 solutions with reference concentrations, then the distribution of MMP-9 concentrations was analyzed according to the 5-scale grading system. Both intraobserver and interobserver reliability was excellent. The readout grades were significantly correlated with the quantified colorimetric densities. The interobserver variance of readout grades had no correlation with the severity of the measured densities. The band density continued to increase up to a maximal concentration (i.e. 5000 ng/mL) according to the calibration curve. The difference of grades reflected the change of MMP-9 concentrations sensitively, especially between grade 2 and 4. Together, our data indicate that the subjective 5-scale grading system in the point-of-care MMP-9 immunoassay is an easy and reliable method with acceptable accuracy.

## Introduction

Dry eye disease (DED) is a multifactorial disease of the ocular surface where various causes collaborate in its pathophysiology^1^. Among them, ocular inflammation is a key factor that composes the pathologic cycle in DED^2^. Tear film hyperosmolarity resulting from unstable homeostasis that fails to protect the eye from desiccating stress triggers inflammatory cascade. This change induces apoptosis of cells that compose an ocular surface where a vicious cycle takes place-further instability of tear film^1^. Considering this close relationship between DED and inflammation, anti-inflammatory therapy was suggested to be effective^3^, and attempts have been made to find a proper and reliable marker for ongoing inflammation level^4^. Recently, matrix metalloproteinase 9 (MMP-9) in tear is a potential marker for inflammation in DED^5–7^.

In eyes with DED, hyperosmolar tear promotes the production of inflammatory cytokines such as interleukin (IL)-1beta and tumor necrosis factor (TNF)-alpha in human limbal epithelial cells through mitogen-activated protein kinase (MAPK) signaling pathway^8^, which proceed to the increase in MMP^9^. MMPs are proteolytic enzymes which is important in wound healing and inflammation. Among those MMPs, MMP-9 has been noticed to play a critical role in degrading epithelial tight junction and results in disruption of barrier function in the corneal epithelium^10^. Although MMP-9 is not directly related to the inflammatory cascade, the indirect connection between MMP-9 and inflammation in DED opens the possibility for clinicians to get hints in deciding their strategies of treatment based on the level of MMP-9 in tear^11^. As such, MMP-9 and MAPK activation and inflammatory cytokine expression in the corneal epithelium may be suppressed by anti-inflammatory treatment including corticosteroid and doxycycline^12^.

Recently, following the improvement of understanding in microscopic biochemistry, researchers are making efforts to detect various molecules such as proteins and metabolites that contains valuable information of human health^13–15^. To keep up with the trend, the point-of-care immunoassay is gaining reputation due to its simplicity, promptness, and cost-effectiveness, compared to previous methods that needs laboratory preparations and disciplined techniques. Moreover, variety of studies are under way to show the result of analysis more effectively, using photothermal^16^, photothermal-thermoelectric^17^, pressure^18,19^, color^20^ or pressure-electrochromic methods^21^. Similarly, for convenient measurement of MMP-9 in tear, the commercially available point-of-care immunoassay was introduced at 2013^6^. It is a disposable and semi-quantitative test that shows the level of MMP-9 with the red-colored band within 10 min after its application to tear film in the inferior conjunctival fornix that applies lateral flow immunoassay (LFIA). This fast and convenient point-of-care device let clinicians get valuable information of ocular surface inflammation and guided them to appropriate treatment at the point of seeing the patients.

However, although it is easy to detect results with bare eyes, the result in color is semi-quantitative that requires reliable grading system that reflects concentration of target material reasonably. Previously proposed dichotomic judgment for the color strip of the MMP-9 immunoassay which is negative versus positive didn’t properly divide the DED profiles by both symptoms and signs^22^. Given that the intensity of the visual test result band correlates with the amount of MMP-9 in the sample^23^, the 5-scale grading system for interpretation has been proposed and showed a significant relationship with ocular discomfort, tear break-up time (TBUT), corneal staining and tear secretion, moreover was useful to monitor treatment response for the topical cyclosporin A^24^.

In this regard, we herein analyzed intraobserver and interobserver reliability of the 5-scale grading system of the point-of-care Immunoassay for tear MMP-9 and validated the accordant 5-step grades using a standard concentration calibration curve to assess whether the system is reliable and reflects the MMP-9 level accurately.

## Results

The mean period of the clinical experience of 6 observers in the field of ophthalmology was 31.33 ± 45.12 months.

### Reliability of the 5-scale grading system of Inflammadry

The repeatability between the two readouts was excellent in all 6 observers, with intraclass correlation coefficients (ICCs) ranging from 0.942 to 0.957. The overall ICCs from all observers between the two readouts was 0.945 (Table 1). Also, the agreement of grading among observers was excellent in both readouts (ICC: 0.953 at 1st readout and 0.943 at 2nd readout, Table 2). The mean value of the readout grades was not significantly different among all 6 observers at both readouts (*P* = 0.067 and 0.076 at the 1st and 2nd readout, respectively).

**Table 1:** Intraobserver reliability in the first and second readouts.

Observer	Grade (1st readout)	Grade (2nd readout)	ICCs for Intraobserver reliability (95% confidential interval)	*P* value
Observer 1	1.440 ± 0.070	1.608 ± 0.075	0.953 (0.938–0.964)	< 0.001*
Observer 2	1.646 ± 0.082	1.349 ± 0.069	0.943 (0.925–0.957)	< 0.001*
Observer 3	1.699 ± 0.076	1.598 ± 0.081	0.954 (0.940–0.965)	< 0.001*
Observer 4	1.617 ± 0.065	1.560 ± 0.069	0.947 (0.931–0.960)	< 0.001*
Observer 5	1.569 ± 0.062	1.622 ± 0.071	0.942 (0.924–0.956)	< 0.001*
Observer 6	1.512 ± 0.081	1.555 ± 0.089	0.957 (0.943–0.967)	< 0.001*
Overall^a^	1.581 ± 0.030	1.549 ± 0.031	0.945 (0.939–0.951)	< 0.001*

Data are shown as mean $\pm$ standard deviation. *P $<$ 0.05.

ICC = Intraclass correlation coefficient.

^a^ICCs for the data set of 1254 total grades (209 images $\times$ 6 observers).

**Table 2:** Interobserver reliability in the first and second readouts.

Readout	ICCs for Interobserver reliability (95% confidential interval)	*P* value
First	0.953 (0.938–0.964)	< 0.001*
Second	0.943 (0.925–0.957)	< 0.001*
Overall^a^	0.989 (0.987–0.991)	< 0.001*

ICC = Intraclass correlation coefficient.

Data are shown as mean $\pm$ standard deviation. *P $<$ 0.05.

^a^ICCs for the data set of 518 total grades (209 images $\times$ 2 readouts).

### Correlation of readout grades with the colorimetric band density

**Figure 1:**
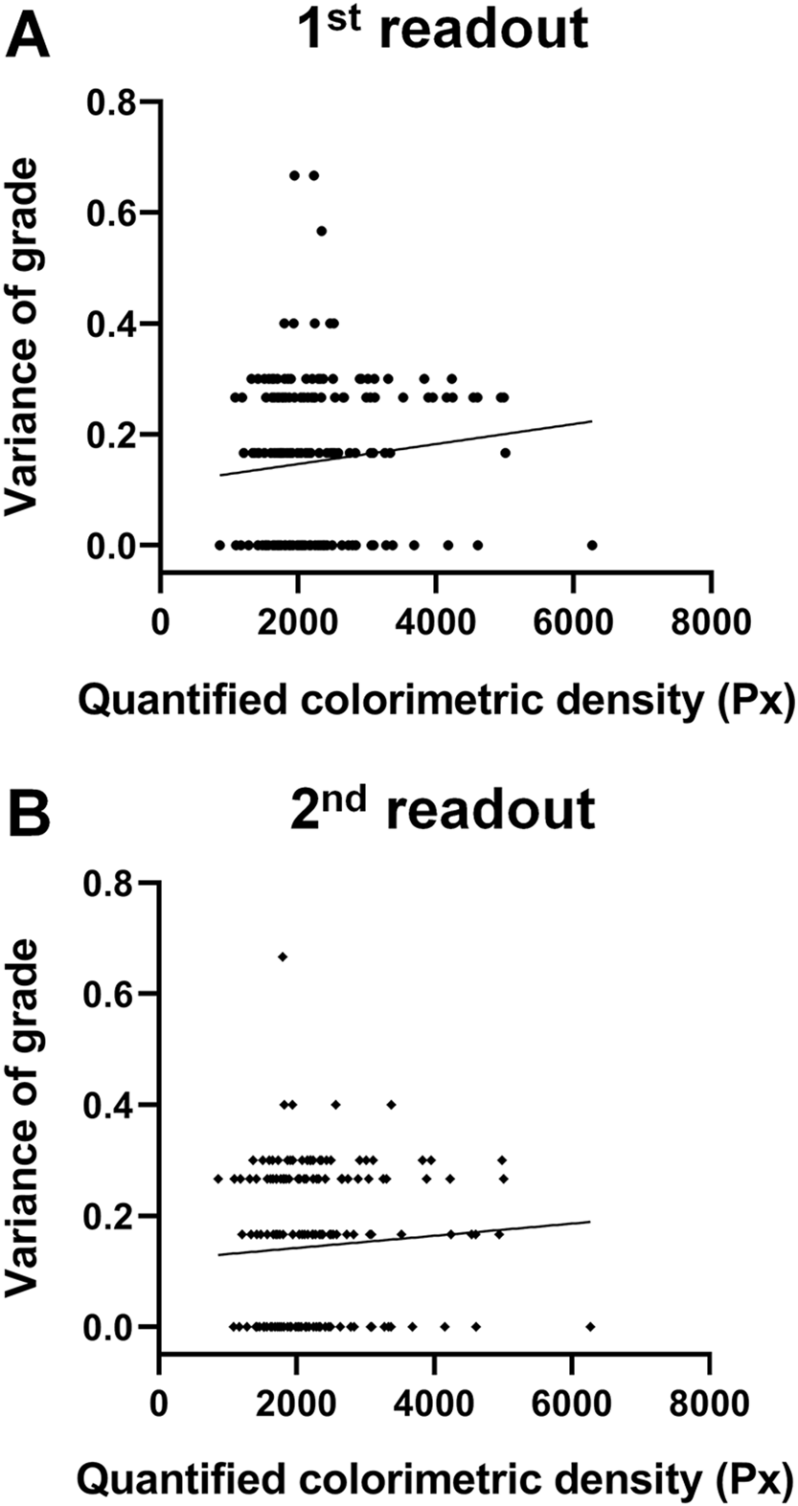
Scatterplots showing the relationship between the variance and the quantified colorimetric densities of the red band of the point-of-care matrix metalloproteinase (MMP)-9 immunoassay at both 1st readout and 2nd readouts. The correlation was not statistically significant at both readouts (*R*^*2*^ = 0.011 and *P* = 0.132 in **A**, *R*^*2*^ = 0.005 and *P* = 0.321 in **B**).

The observer-led readout grades were significantly correlated with the quantified colorimetric band densities in all 6 observers at both readouts (Table 3). There was no correlation between the inter-observer variance of readout grades and the quantified colorimetric densities of the red band at both 1st readout (*R*^2^ = 0.011 and *P* = 0.132) and 2nd readout (*R*^2^ = 0.005 and *P* = 0.321), which indicates that the severity of MMP-9 expression in immunoassay kits did not the affect the readout grades according to the 5-scale grading system (Fig. 1).

**Table 3:** Correlation between the readout grades and the quantified densitometric color in all of 6 observers and in the first and second readouts.

Observer	1st readout		2nd readout	
	r coefficient	P value	r coefficient	P value
Observer 1	0.612	$<$ 0.001*	0.599	$<$ 0.001*
Observer 2	0.611	$<$ 0.001*	0.621	$<$ 0.001*
Observer 3	0.596	$<$ 0.001*	0.592	$<$ 0.001*
Observer 4	0.580	$<$ 0.001*	0.612	$<$ 0.001*
Observer 5	0.557	$<$ 0.001*	0.613	$<$ 0.001*
Observer 6	0.588	$<$ 0.001*	0.579	$<$ 0.001*
Overall^a^	0.587	$<$ 0.001*	0.597	$<$ 0.001*

*P $$<$$ 0.05.

^a^Values for the data set of 1254 total grades (209 images $\times$ 6 observers) at each readout.

### Correlation curve between the colorimetric band density and MMP-9 concentration

**Figure 2:**
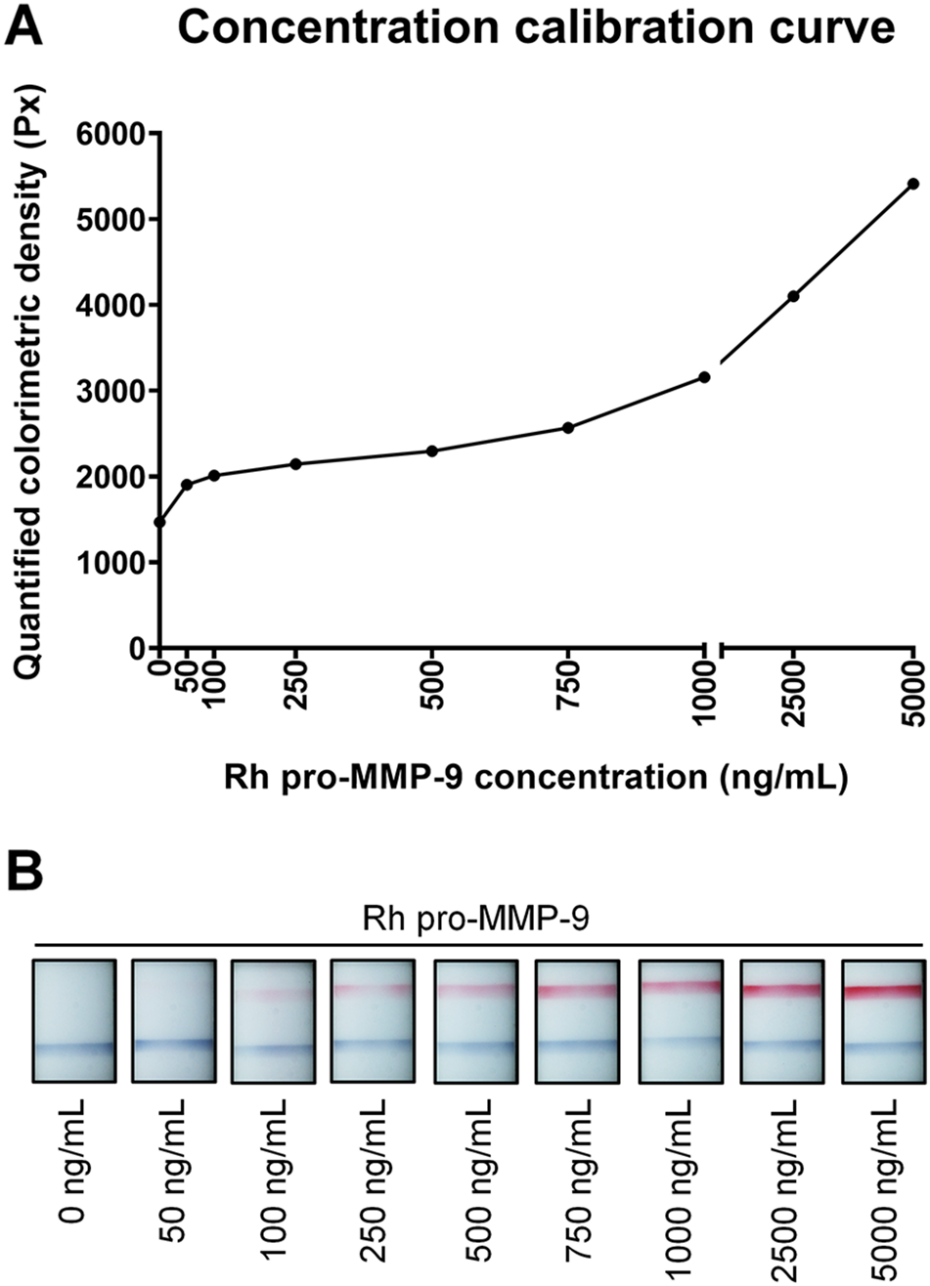
Standard calibration curve showing the concentration-band density relationship using various concentrations of recombinant human (rh) pro-matrix metalloproteinase (MMP)-9 solutions. (**A**) There are slow progression sections below 100 ng/mL and over 750 ng/mL of concentration while there is a slow progression section between concentrations of 100 ng/mL and 750 ng/mL. The values in each concentration is an average value from 3 independent samples of the same concentration. (**B**) Representative photographs of readout windows of the point-of-care matrix metalloproteinase (MMP)-9 immunoassay at the different concentrations of rh MMP-9 from 0 ng/mL to 5000 ng/mL.

We made standard concentration calibration curve using the 9 different concentrations of MMP-9 solutions (Fig. 2). The curve has steep concentration-gradient progression below the concentration of 100 ng/mL and over 750 ng/mL and has slow progression between the concentration of 100 ng/mL and 750 ng/mL. There was no revealed plateau up a maximal concentration (i.e. 5000 ng/mL).

### Validity of the 5-scale grading system of inflammadry

The quantified colorimetric band densities were significantly different among all grades. Although the concentration of the recombinant human (rh) pro-MMP-9 was positively correlated with the 5-scale grades (*R*^2^ = 0.623 and *P* $<$ 0.001), the 1-grade step change could not reflect the difference of MMP-9 concentration at lower ranges of grades between grade 0 and 2 (Table 4). On the other hands, the difference of grades in higher ranges of grades between grade 2 and 4 detected the change of MMP-9 concentration sensitively (Table 4).

**Table 4:** Difference of values of recombinant human pro-matrix metalloproteinase 9 (MMP-9) concentration and quantified colorimetric density according to the readout grades based on the 5-scale grading system.

Grade	N	Rh pro-MMP-9 concentration (ng/mL)			ANOVA P value	post hoc test P value^a^				
		Mean $\pm$ SD	Range	Median		vs Grade 0	vs Grade 1	vs Grade 2	vs Grade 3	Vs Grade 4
Grade 0	30	20.0 $\pm$ 24.9	0–50	0	$<$ 0.001*	–	$>$ 0.999	0.007*	$<$ 0.001*	$<$ 0.001*
Grade 1	37	217.6 $\pm$ 193.7	50–750	100		–	–	0.137	$<$ 0.001*	$<$ 0.001*
Grade 2	42	632.1 $\pm$ 411.2	100–2500	500		–	–	–	$<$ 0.001*	$<$ 0.001*
Grade 3	28	1607 $\pm$ 1233	250–5000	1000		–	–	–	–	$<$ 0.001*
Grade 4	25	4100 $\pm$ 1225	2500–5000	5000		–	–	–	–	–

Grade	N	Quantified colorimetric density (Px)			ANOVA P value	post hoc test P value^a^				
		Mean $\pm$ SD	Range	Median		vs Grade 0	vs Grade 1	vs Grade 2	vs Grade 3	Vs Grade 4
Grade 0	30	1636 $\pm$ 225.5	1420–2031	1550	$<$ 0.001*	–	0.002*	$<$ 0.001*	$<$ 0.001*	$<$ 0.001*
Grade 1	37	2081 $\pm$ 152.7	1880–2523	2080		–	–	0.020*	$<$ 0.001*	$<$ 0.001*
Grade 2	42	2412 $\pm$ 298.7	1970–3520	2413		–	–	–	$<$ 0.001*	$<$ 0.001*
Grade 3	28	3456 $\pm$ 843.9	2138–5496	3520		–	–	–	–	$<$ 0.001*
Grade 4	25	5075 $\pm$ 608.9	3847–5634	5106		–	–	–	–	–

SD = Standard deviation.

*P $$<$$ 0.05.

^a^Bonferroni’s post hoc.

## Discussion

In this study, we presented the reliability and validity of the 5-scale grading system of point-of-care MMP-9 immunoassay. We found that both intraobserver and interobserver reliability of the observer-led subjective grading according to the 5-scale grading system were excellent. The 5 steps of severity grades were significantly parallel with the quantified color densities of the red bands. The colorimetric density of results band increased up to 5000 ng/mL of rh pro-MMP-9 which was a maximal concentration. Although the 1-grade step difference in lower ranges between grade 0 to 2 according to the 5-scale grading system did not distinguish the difference of MMP-9 concentration significantly, the difference of grades in higher ranges between grade 2 to 4 reflected the change of MMP-9 concentration well.

Although MMP-9 has been suggested as an excellent indicator for inflammation in DED^5,25^, its usage had not been easy in actual clinical circumstances. Previously, several studies reported the effect of complex biochemical tools such as MMP activity assay kit^5^, real-time polymerase chain reaction for RNA level of expression^5,10,12^, double sandwich enzyme-linked immunosorbent assay kit^25^ or SDS-gelatin polyacrylamide gel electrophoresis^10,12,25^. These techniques are quite accurate but were limited to experimental use due to the high cost, the time required, and the inconvenience.

Recently, InflammaDry was released to cover these shortcomings. InflammaDry applies lateral flow immunoassay (LFIA) method for detection of MMP-9, which is nowadays widely used in various fields as a point-of-care device thanks to its promptness, convenience and low-price. It is a paper system where antibodies are used for recognition elements. The absorbed tear sample meets the buffer that prepares the sample to be suitable for detection and goes up through the paper by capillary action. Along the way, it passes through the conjugate release pad which contains specific antibodies for the target analyte. The antigen–antibody complex then migrates to detection zone and results in a test band next to the control band^26^.

It is easy to use and takes only a few minutes to get results, opening the possibility of convenient detection of MMP-9 level as point-of-care testing. Like many other easy-to-use and cost-effective point-of-care immunoassays, InflammaDry attracts clinicians in that it detects MMP-9 level in tear and it offers useful information of the ocular surface. As MMP-9 is secreted into tear as a side-product of ocular surface inflammation, it provides hints of current inflammatory status of the eye, when to start anti-inflammatory treatment and judgement of the result of treatment^10–12,27^. In non-qualitative tests such as InflammaDry, intuitive grading system that reflects the target analyte concentration and that shows proper agreements among interpreters should be prepared to be widely used. Because InflammaDry has high sensitivity and specificity and high agreement with other diagnostic tools for DED^6,7,11,24^, it is now accepted as one of the favorable test modalities for DED.

The range of clinical experience of the enrolled observers was widely distributed from 2 to 122 months. Even untrained or undertrained clinicians could have interpreted the red expression band very easily with excellent reliability. This was probably due to the intuitive but precise process of a 5-scale grading system, showing how easily it can be applied in clinics. Also, our study gives evidence that this test can be reliably conducted worldwide only with distributed standard photographs.

In a commonsense way, the darker red bands are easy to be identified, thus may be easily and rapidly classified into grade 4 (i.e. positive) or grade 5 (i.e. strong positive). While the pale or weak red bands which are unevenly reactive were occasionally confusing and some observers appealed difficulty to give a grade among grade 0 (i.e. negative), 1 (i.e. trace), and 2 (i.e. weak positive). In a similar vein, a previous study that evaluated the reliability of 4 clinical grading systems for corneal staining revealed that the interobserver variance of grades increased with the corneal staining score^28^. In fact, in our study, the interobserver reliability of the subjective grading was not interrupted by the color density of the red band, although this issue should be clarified through a future study with a larger number of observers and objects.

According to a concentration calibration curve made in this study, the progression was still steep up to a maximal concentration (i.e. 5000 ng/mL) unlike a previous study showed a plateau of band density after the concentration of 500 ng/mL of activated MMP-9^23^. Although it is hypothetical, we think that our use of pro-MMP-9 might decrease the sensitivity of InflammaDry. Unfortunately, the 1-grade step change from grade 0 to 2 did not reflect the difference of MMP-9 concentration significantly while the 1-grade step change from grade 2 to 4 distinguished the MMP-9 concentration well. In a similar vein, there was a section of slow progression between a concentration of 100 ng/mL and 750 ng/mL in the calibration curve. Because the curve was not linear as also seen in a study by Bang et al.^23^, it is thought that InflammaDry does not detect the change of MMP-9 sensitively especially at the range of lower concentrations. Therefore, interpreters should pay more attention when they see light red bands in readout windows. We think that a novel 4-scale grading system of which grade 1 is a combination of grade 1 and 2 in the 5-scale grading system may be an alternative to improve the validity. But this would rather be confirmed with meticulous evaluation in a future study using a large number of samples.

This study has several limitations. Although InflammaDry detects both inactive and active form of MMP-9, only the recombinant pro-MMP-9 was used in experiments to make prepared solutions which might be different in detailed characteristics from activated human MMP-9 in vivo. In addition, the range of MMP-9 concentrations in each grade was quite wide probably due to a small number of trials. The quantified colorimetric density of red bands from 0 ng/mL MMP-9 solutions was not 0 but was over 1400. Despite it might be a noise signal lowering the accuracy of quantification of color density using Image*J* software, we rather think that the detected densities from 0 ng/mL of MMP-9 solutions were because we did not extract red color from photographs to reflect a practical circumstance for interpretation under visible light.

In fact, the clinical application of 5-scale grading system may be significantly limited because the manufacturer of InflammaDry clearly recommends in its manual that observers interpret results in a dichotomous manner (i.e. negative vs. positive). It implies that the analysis of tear MMP-9 using InflammaDry is more like qualitative than quantitative or semi-quantitative. Despite we revealed the probable correlation of colorimetric densities in InflammaDry with MMP-9 levels experimentally, clinicians should keep in mind that the semi-quantitative approach using the 5-scale grading system is not certified by the manufacturer. Therefore, we think that clinicians should be very cautious about determining the gradient of MMP-9 in tear based on the density gradient of red lines in InflammaDry when they meet dry eye patients and establish therapeutic strategies using the results of InflammaDry.

Nevertheless, our study has strength. This is the first report to validate the 5-scale grading system of point-of-care immunoassay device, InflammaDry. Not only it would advise clinicians in interpreting the information concerning inflammatory status of ocular surface and making clinical decisions, but also would be a precedent of evaluating validity and reliability of point-of-care devices with colorimetric result windows. As visual detection is intuitive, timesaving and therefore is one of the promising ways to demonstrate result, similar research would help establishing the standard for evolving point-of-care devices.

In conclusion, our results indicate that the subjective 5-scale grading system in the point-of-care MMP-9 immunoassay is an easy and reliable method with acceptable accuracy.

## Methods

This study was a retrospective observational study in conjunction with in vitro experiments without rh MMP-9 solutions, of which the whole process properly followed the tenets of the Declaration of Helsinki. It was also approved by the Chung-Ang University Hospital Institutional Review Board. The obtainment of informed consents was waived off by institutional review board due to the retrospective nature of the study.

### Assay protocol of InflammaDry from tear

The immunoassay test for MMP-9 was performed according to the manufacturer’s instructions for use and previously well-established^6,7^. Brief, the operator gently dabbed the sample collector of a test kit in multiple locations along the palpebral conjunctiva until the sampling fleece was saturated with tears. Next, the sample collector is assembled to the test cassette, then the test pad is dipped into a buffer solution for 20 s for activation. Last, after 10 min, the red test band in a readout window was read.

### Experimental evaluation of recombinant human MMP-9 solutions using InflammaDry

The rh pro-MMP-9 was purchased form BioLegend® (550502, San Diego, CA, USA) and diluted with phosphate buffered saline to produce 0, 50, 100, 250, 500, 750, 1000, 2500, 5000 ng/mL of MMP-9 solutions. 20μL of each sample was applied to InflammaDry sample collector with micropipette. After assembling the sample collector with test cassette as manufacturer’s instruction, it was soaked with 300 μL of enclosed buffer solution. After 20 min, photographs of result window were taken under same illuminance. The same independent procedure was done for each concentration for 3 times.

### Photo-documentation of InflammaDry results

We used 209 anonymous photo results of the point-of-care MMP-9 immunoassay (InflammaDry, Quidel, San Diego, CA, USA) from DED patients who visited our institute and 27 photo results from rh pro-MMP-9 samples which were experimentally diluted at diverse concentrations. We took photographs of detector windows of kit, then saved them in jpg image files. Every photograph was taken with the same digital camera under the stereo microscope (SZ61, Olympus, Tokyo, Japan) at the same place, the same person, and the same illumination at the time of point-of-care testing. There was no patient-identifiable information in every image.

### Quantitative colorimetric analysis of InflammaDry results

**Figure 3:**
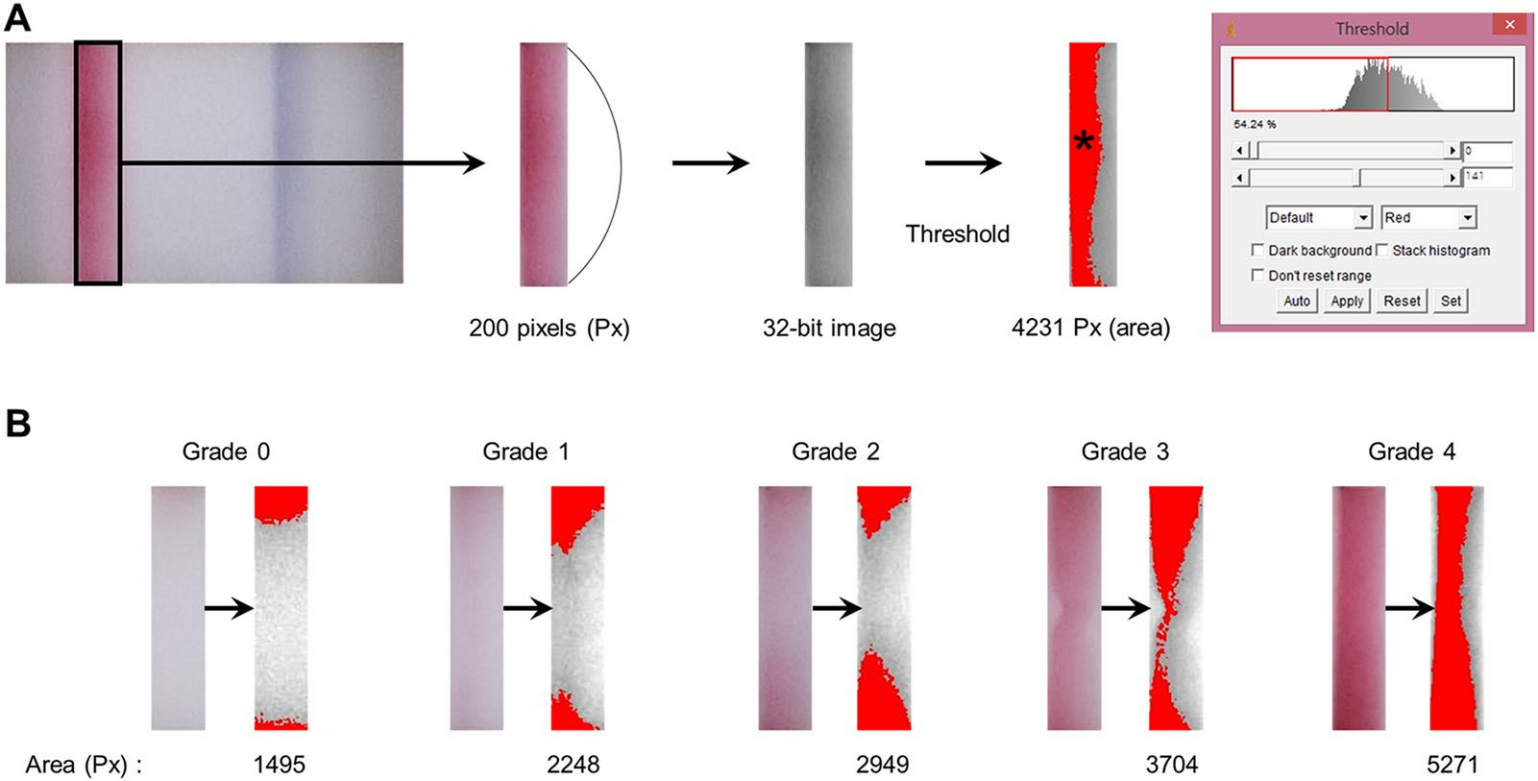
Representative images showing the processes for the quantification of the colorimetric density of the red band in the readout window of the point-of-care matrix metalloproteinase (MMP)-9 immunoassay. (**A**) First, the MMP-9-expressing red band was outlined with a black rectangle, then was cropped. The vertical length was sized into 200 pixels (Px). The image was converted to the 32-bit one and the area with relatively high color density was detected by auto-threshold. The red-colored area (asterisk) was estimated. (**B**) The representative 5 images in each grade before and after the color threshold.

The densities of the red test band for MMP-9 expression using Image*J* software (National Institutes of Health; http://rsbweb.nih.gov/ij/). First, the photos used for estimation were opened in Image*J*, then the red test bands were rectangularly cropped along to the edge of the red stripe. The cropped ones were processed into a 32-bit image and were adjusted into equal length (200 pixels [Px] in height with a constrained aspect ratio). Then, we measured the estimated area (square Px) of the red-colored band by thresholding (default mode with red expression) (Fig. 3).

### Grading of InflammaDry results by the observer

**Figure 4:**
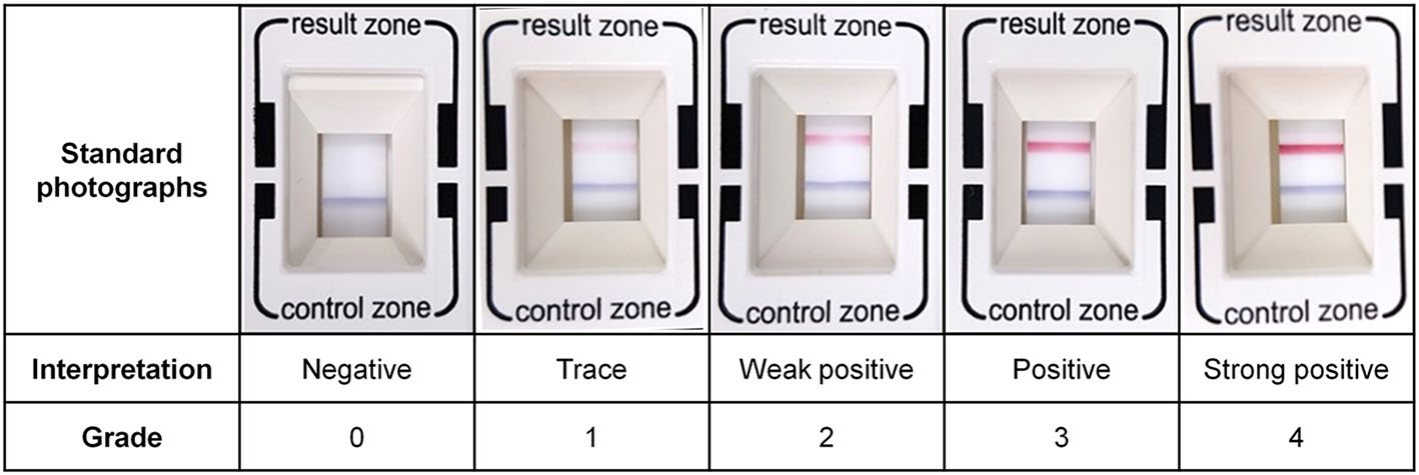
Standard photographs for 5-scale grades ranged from 0 to 4 along to the color density of the red band in the readout window of the point-of-care matrix metalloproteinase (MMP)-9 immunoassay.

Six ophthalmologists (1 staff and 5 trainees) participated as observers in grading 209 images of point-of-care MMP-9 immunoassay from tear samples and in grading 27 images from rh pro-MMP-9 solutions. Amongst, observer 4 and 5 had never experienced the readout of point-of-care MMP-9 immunoassay. Density evaluation of the red test band was performed based on the 5-scale grading system composed of 0 (i.e. negative), 1 (i.e. trace), 2 (i.e. weak positive), 3 (i.e. positive) and 4 (i.e. strong positive)^24^, and the relevant standard photograph showing each grade (Fig. 4) was given to the observer for readout. The images were cropped with the margin justified to a readout window of the test cassette. Each observer graded images in an independent space and they never met to discuss the study.

### Assessment of reliability of the 5-scale grading system

The readout using total 209 photographs of results from tear was performed two times with a 2-week interval to verify the intraobserver and interobserver reliability of the point-of-care MMP-9 immunoassay. Next, to determine the degree of interobserver agreements according to the severity of tear MMP-9 expression, the relationship between the variance of readout grades according to the 5-scale grading system and the values of quantified colorimetric densities was analyzed using the linear regression analysis.

### Assessment of validity of the 5-scale grading system

The color density of each result was measured with Image*J* and the mean value of the density were calculated for each concentration to draw a standard concentration calibration curve. Random numbers were assigned to total 27 photo-results of the point-of-care MMP-9 immunoassay from 9 different concentrations of rh pro-MMP-9 and 6 ophthalmologists were asked to interpret the results according to the 5-scale grading system. Difference of the quantified colorimetric density values and the concentrations of rh pro-MMP-9 according to grades were analyzed using the 162 pooled readouts (i.e. 27 results $\times$ 6 observers).

### Statistical analysis

The data are presented as the mean ± standard deviation. Statistical analyses were performed using the SPSS software version 20.0 (SPSS, Inc., Chicago, IL, USA) and GraphPad Prism v.8.1.2 (Graph-Pad Software, La Jolla, CA, USA). Intraobserver and interobserver reliability were assessed with intraclass correlation coefficients (ICCs). The correlation between the quantitative density of the red band and the observer-led subjective grades was analyzed using the Pearson correlation test and linear regression analysis. To compare the values among more than two groups, the data were analyzed using one-way ANOVA followed by Bonferroni’s *post-hoc* test. *P* values < 0.05 were considered statistically significant.
